# Guillain-Barré Syndrome Induced by Vaccination Against COVID-19: A Systematic Review and Meta-Analysis

**DOI:** 10.7759/cureus.37578

**Published:** 2023-04-14

**Authors:** Olajide Bamidele Ogunjimi, Gabriela Tsalamandris, Antonella Paladini, Giustino Varrassi, Panagiotis Zis

**Affiliations:** 1 Medical School, University of Sheffield, Sheffield, GBR; 2 Medical School, University of Cyprus, Nicosia, CYP; 3 Anesthesiology, University of L'Aquila, L'Aquila, ITA; 4 Pain Medicine, Paolo Procacci Foundation, Rome, ITA; 5 Neurology, University of Cyprus, Nicosia, CYP

**Keywords:** guillain-barré syndrome, covid-19 vaccination, covid-19, post vaccination complications, immune mediated complications

## Abstract

Guillain-Barré syndrome (GBS) is a rare but serious immune-mediated neurological condition characterized by damage to the peripheral nervous system. Two-thirds of cases of GBS are diagnosed following infection; however, vaccination has also been linked to GBS pathogenesis. The aim of this systematic review and meta-analysis was to establish the prevalence of GBS following vaccination against the SARS-CoV-2 virus, which causes COVID-19, describe the clinical and neurophysiological characteristics, and identify potential determinants. A systematic review of the literature regarding post-vaccination GBS was conducted using the PubMed database. Seventy papers were included. The pooled prevalence of GBS after vaccination against COVID-19 per has been established to be 8.1 (95% CI 30-220) per 1,000,000 vaccinations. Vaccination with vector vaccines - but not mRNA - has been associated with an increased risk of GBS. More than 80% of the patients developed GBS within 21 days following the first dose of the vaccination. The interval between the vaccination and GBS was shorter in patients who were vaccinated with mRNA versus vector vaccines (9.7±6.7 days versus 14.2±6.6 days). Epidemiological findings regarding post-vaccination GBS revealed a higher prevalence in males and people between the ages of 40 and 60 years, with a mean age of 56.8±16.1 years. The most common type was the acute inflammatory demyelinating polyneuropathy type. Most cases responded well to treatment. In conclusion, vaccination against COVID-19 with vector vaccines seems to increase the risk of GBS. GBS occurring following vaccination does differ in characteristics from GBS during the pre-COVID-19 era.

## Introduction and background

From the discovery of a new SARS-CoV-19 single-stranded RNA virus that causes coronavirus disease in 2019 (COVID-19) and the declaration of the COVID-19 pandemic in March 2020 till today, numerous confirmed COVID-19 cases and deaths have been reported. Worldwide research and development took place, resulting in the development of various vaccines against the virus.

There are four main categories of anti-SARS-CoV-2 vaccines, namely the whole virus, protein subunit, nucleic acid piece, and the viral vectors vaccine. These vaccines generally work by initiating immune reactions to the spike proteins on the surface membrane of SAR-CoV-2, thus providing immunity within the community as well as against the severe form of the disease. Various symptoms have been associated with the COVID-19 vaccine and are usually mild and self-limiting. There have been reports, however, of serious reactions, ranging from mild to severe hypersensitivity reactions, as well as numerous cases of neurological complications, including Guillain-Barré syndrome (GBS) [1].

GBS is a rare but serious immune-mediated disease characterized by damage to the peripheral and autonomic nervous system and is one of the known leading causes of flaccid paralysis worldwide [2], with an estimated annual median incidence of 11 persons per 1,000,000 population [3]. Although it is relatively rare, it may be life-threatening and debilitating.

The clinical manifestations of GBS usually reach their peak within four weeks of onset and include progressive ascending limb weakness and profound areflexia, while symptoms, such as paresthesia and/or pain, may occur in a few cases [3]. Autonomic dysfunction, respiratory failure, and, less commonly, cranial neuropathies are other symptoms associated with GBS.

Nerve conduction studies (NCS) are extremely useful in supporting the diagnosis of GBS and in differentiating between the numerous phenotypes. The electrodiagnostic patterns seen in GBS can be classified as Acute Inflammatory Demyelinating Polyneuropathy (AIDP), acute motor axonal neuropathy (AMAN) or motor and sensory axonal neuropathy (AMSAN) or Miller-Fisher syndrome (MFS), according to their respective clinical manifestations and findings derived from NCS [3-6]. GBS is treated with intravenous immunoglobulin infusion or plasma exchange, depending on the severity of the symptoms, and the outcome is generally favorable [5].

The aim of this study is to systematically review the current literature to establish the prevalence, severity, and determinants of GBS in COVID-19 vaccine recipients.

## Review

Methods

Literature Search Strategy

A systematic search was conducted using the PubMed database on May 23, 2022 using three Medical Subject Headings terms. Term A was “vaccine OR vaccinated,” Term B was “COVID-19 OR SARS-CoV-2 OR coronavirus,” and Term C was “GBS OR Guillain-Barré syndrome OR acute demyelinating polyneuropathy OR acute motor axonal neuropathy OR acute sensorimotor polyneuropathy OR Miller-Fisher syndrome OR AIDP OR MFS OR AMAN OR AMSAN”. English language filter was applied. For completeness, any results from ongoing or unpublished trials were searched for at http://www.clinicaltrials.gov/. All study data were aggregated and referenced in accordance with Preferred Reporting Items for Systematic Reviews and Meta-analysis (PRISMA) guidelines.

Inclusion and Exclusion Criteria

Articles eligible for inclusion in this review had to meet the following criteria: papers containing information about human subjects who had received any type of vaccination against COVID-19 and who subsequently developed clinically confirmed GBS.

The exclusion criteria for this review include (i) non-human studies, (ii) duplicate studies or studies referring to the same populations, and (iii) nonoriginal articles (review, medical hypothesis, letter to the editor, etc.).

In consecutive order, title screening, abstract screening, full-text screening, and reference screening, were implemented to filter out non-relevant papers. This left relevant articles suitable for inclusion. The papers, which were eligible for inclusion, were decided upon by two researchers. Reference screening was implemented using Google Scholar for the sole use of identifying papers that may have been missed during the original search; however, no additional literature was yielded.

Data Extraction

Data from the included literature were extracted and recorded using an Excel spreadsheet. Data collected included the title; the name of the author; year of publication; demographics such as the age of subjects (years), gender, the prevalence of GBS after vaccination, time of onset of clinical symptoms of GBS after vaccination, number of vaccines received, type of vaccine administered (AstraZeneca, Moderna, Pfizer, Johnson & Johnson or others) and laboratory data such as covid serological screen, specific antibody titers (such as anti-ganglioside antibody titer, and anti-GMQ1e.t.c), cerebrospinal fluid (CSF) analysis (protein and cell counts), electromyography and NCS’s findings (axonal or demyelinating), treatment received, outcome of treatment and any disability or life-changing events.

Synthesis of Results and Statistical Analyses

Aggregated data were used where possible. Statistical pooled proportion calculations conducted in R language, used default settings of the “meta” package and “metaprop” function (random effects model) [7]. Each meta-analysis presented the I² statistic and forest plots, which evaluated heterogeneity [8]. Variation can be suggested as study heterogeneity or chance using this statistic. Negative I² values are put equal to 0, and values range between 0% and 100% [8]. Heterogeneity can be quantified as low, moderate, and high, with upper limits of 25%, 50%, and 75% for I², respectively [8]. Where data did not lend itself to meta-analysis, a narrative approach was taken.

Frequencies and descriptive statistics were examined for each variable. Comparisons between two groups were made using Student’s t-tests for normally distributed continuous data and chi-square test for categorical data. Analyses between more than two groups were made using one-way analysis of variance for continuous data and chi-square test for categorical data. Bonferroni’s correction for multiple comparisons was applied as appropriate. Level of significance was set at 0.05.

Compliance With Ethical Guidelines

As this is a systematic review based on the existing literature regarding post-covid vaccination GBS, there was no need to conduct an ethics review.

Results

Study Characteristics

**Figure 1:**
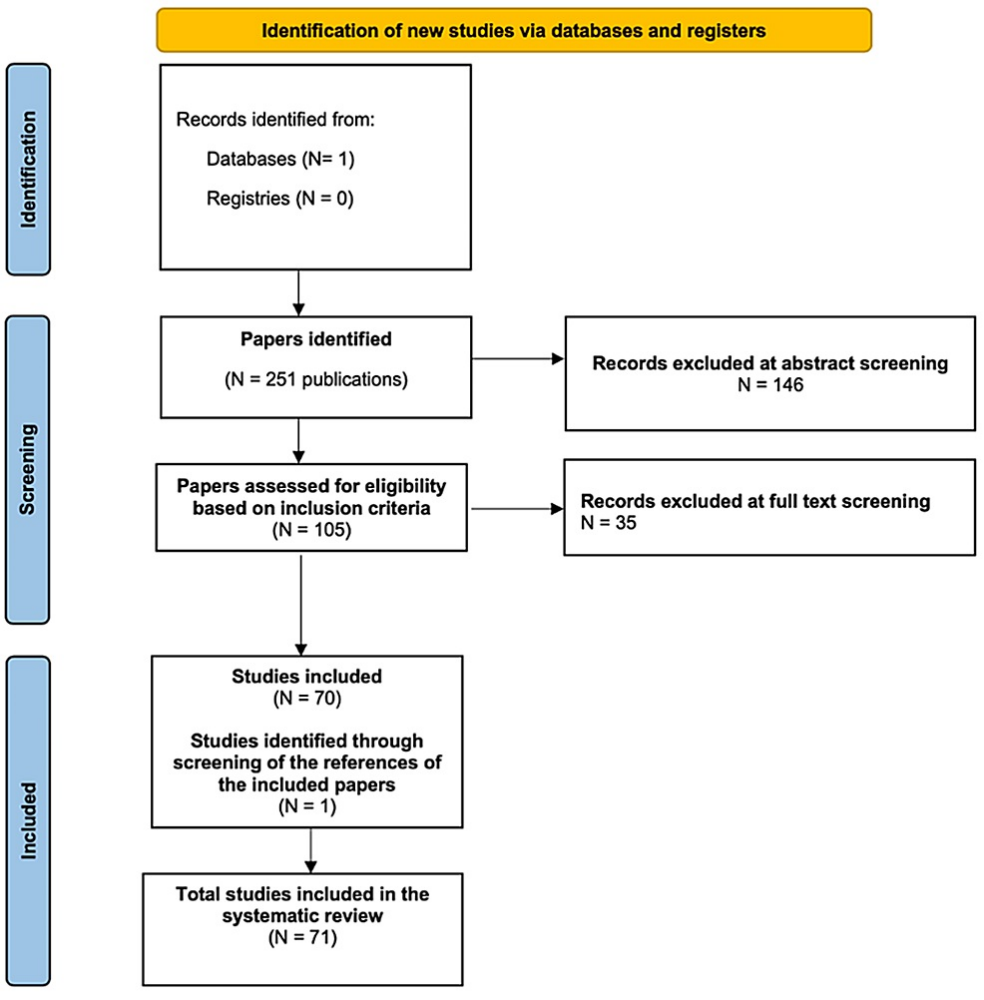
PRISMA flow diagram of the study.

The above-mentioned research strategy yielded 251 studies. Following abstract screening, 146 studies were excluded. After evaluation of the full texts of the remaining 105 reports, 36 more publications were excluded as they did not meet the outlined inclusion criteria. One paper which was eligible for inclusion was identified through the reference lists of the included papers. In total 71 articles have been included in the review [1,2,6,9-76]. Three of those papers provided relevant data regarding the prevalence and incidence of post COVID-19 vaccination GBS [9-11], while the others were mainly case reports or small case series comprising of a total of 138 patients with COVID-19 vaccine-related GBS. The PRISMA chart in Figure 1 shows the outcome of the selection process at each stage of the procedure.

Prevalence of GBS Following COVID-19 Vaccination

Meta-analysis of the three studies which contained prevalence data was performed. In their study, Patone et al. [9] showed that in England, there was an overall increased risk of GBS with an incidence rate ratio (IRR) of 2.9 (95% confidence interval 2.2-3.9) 15-21 days after vaccination. Interestingly, 3.8 per million excess cases of GBS were estimated in subjects who received the Astrazeneca vaccine in the 1-28-day risk period. However, the authors did not observe an increased risk of GBS in those who received the Pfizer vaccine. Similarly, in Kerala, India, Maramattom et al. reported that following vaccination with the Astrazeneca vaccine the frequency of GBS was 1.4- to 10-fold higher than what expected in the same period [10] and Osowicki et al. reported the frequency of GBS subsequent to vaccination with the Astrazeneca vaccine to be 1.6-fold higher than expected [11].

**Figure 2:**
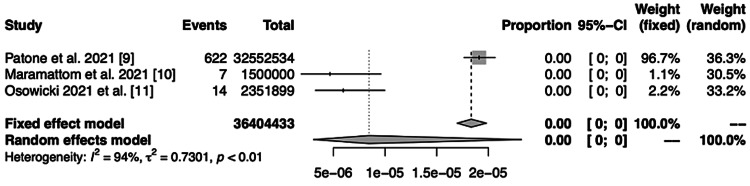
Forest plot showing outcome of meta-analysis of prevalence GBS following COVID-19 vaccination.

**Figure 3:**
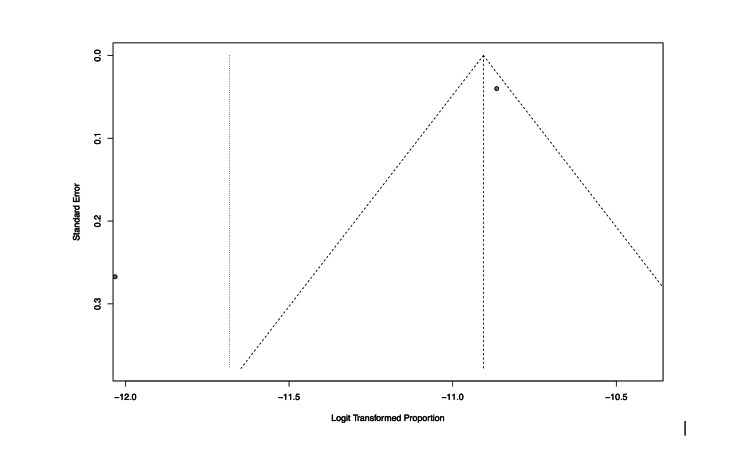
Funnel plot showing high heterogeneity in the data and outcome of meta- analysis of prevalence of post covid-vaccination GBS.

The pooled prevalence of GBS after vaccination against COVID-19 has been established to be 8.1 (95% CI 30-220) per 1,000,000 vaccinations (Figure 2). However, as shown in the respective funnel plot (Figure 3) there was high heterogeneity across these studies (I^2^ = 93.7%).

Characteristics of Gbs Patients Following COVID-19 Vaccination

Type of vaccines: Most cases received the Astrazeneca vaccine (56%), followed by Pfizer (20%), J&J (7%), Sputnik (7%), Moderna (5%), Sinovac (4%) and Novavax (1%). GBS occurred after receiving the first dose of the vaccination (where applicable) in 110 cases (80%).

Demographics: In total, 138 cases of GBS following the COVID-19 vaccination were recorded, of which, 82 (59.4%) occurred in males while 56 (40.6%) were in females. The highest number of cases was reported in the age group 41-60 years and the mean age of patients was found to be 56.8±16.1 years.

Time interval: The mean interval between vaccination and GBS development was 13.0±6.9 days, ranging from one to 37 days. More than 90% of GBS cases developed three weeks post-vaccination. Table 1 summarizes the demographic characteristics and the time interval of GBS development and the CSF findings per type of vaccine.

**Table 1:** Summary of the demographic characteristics and the time interval of GBS development and the CSF findings per type of vaccine.

	Astrazeneca (n=77)	Pfizer (n=28)	J&J (n=9)	Moderna (n=7)	Novavax (n=1)	Sputnik (n=9)	Sinovac (n=6)
Age, in years (SD)	57.9 (12.7)	56.4 (22.1)	48.1 (11.9)	68.2 (13.9)	76	50.7 (15.4)	51.7 (25.0)
Interval, in days (SD)	13.9 (6.3)	9.8 (6.1)	14.7 (5.9)	9.5 (9.5)	8	16.3 (6.0)	13.7 (12.2)

CSF findings: There was found to be an elevated CSF protein level in 87 (96%) of the 91 patients on which a lumbar puncture was performed while 4 patients had low to normal CSF protein levels. The mean reported value of CSF protein level was 221mg/dL.

Neurophysiological findings: Out of the 87 cases with reported NCS and EMG findings, 57 (66%) of the patients presented with demyelinating polyneuropathy while 23 (26%) had axonal phenotype GBS. Other forms included isolated bilateral facial nerve palsy and MFS. There was no significant difference in patient demographics or clinical characteristics when comparing demyelinating and axonal GBS groups.

Comparison of Vector and mRNA Vaccines

Comparison between 101 patients who received a vector vaccine (Astrazeneca, J&J, Sputnik, Sinovac) and 35 patients who received an mRNA vaccine (Moderna, Pfizer) was performed. Novavax is a different technology and thus, was not included in this pooled analysis.

The two groups did not differ significantly regarding age (56.0±14.0 versus 58.5±21.2, p=0.440). A trend of statistical significance was noted regarding gender as more males were found among the patients who developed GBS following a vector vaccine (64% versus 46%, p=0.053). This trend disappeared during the secondary analysis which only included the patients that developed GBS following the first dose of vaccinations requiring two doses (64% versus 52%, p=0.315).

The time interval of GBS manifestation was significantly lower in mRNA vaccine recipients (9.7±6.7 days versus 14.2±6.6 days, p < 0.001). A secondary analysis including only patients that developed GBS following the first dose of vaccinations requiring two doses was performed between the 88 patients who received a vector vaccine and 22 patients who received an mRNA vaccine. The time interval between vaccination and GBS was still significantly lower in mRNA vaccine recipients (11.1±6.8 days versus 14.2±6.4 days, p=0.048). No differences between CSF findings (number of cells, protein levels) where found between the groups.

Treatment Outcome and Severity of Gbs in Vaccine Recipients

Information about treatment and outcome was available for 127 patients. Most patients received intravenous immunoglobulins (79.5%), followed by plasma exchange (11.8%). A small minority (8.7%) presented with mild symptoms only and were managed conservatively. One in five patients (20%) required intensive care admission. The only death that was reported was due to autonomic dysfunction.

Most of the affected patients recovered to varying extents and at different rates. The activity level at final follow-up varies widely in each case, ranging from full recovery to persistent on-going motor and facial weakness, ataxia, quadriplegia, extreme pain, and other debilitating symptoms.

Discussion

One of the key findings of this systematic review and meta-analysis is that the prevalence of GBS related to vaccination against COVID-19 is 8.1 (95% CI 30-220) per 1,000,000 vaccinations. This is significantly higher to the epidemiology of GBS in the general population as previous literature suggests that the annual incidence of GBS follows a rising pattern with increasing age, from six (in children) to 27 (in elderly patients of more than 80 years of age) per million per year [3]. All three studies that provide big data about the epidemiology of GBS following vaccination against COVID-19 show that the majority of cases concern vaccination with a vector vaccine. In fact, Patone et al. [9] did not observe an increased risk of GBS in those who received a mRNA vaccine.

Secondly, our analysis showed that the time interval between vaccination and GBS was much lower for mRNA than for vector vaccines. The different technology and resultant immune-mediated mechanisms may account for this finding. However, these figures should be interpreted with caution given the fact that a true association between development of GBS following vaccination with an mRNA vaccine may not exist.

Thirdly, the most common neurophysiological type of GBS secondary to vaccination against COVID-19 was the demyelinating type (AIDP), a finding which agrees with the literature on GBS [77]. Finally, the outcome of GBS following vaccination against COVID-19 did not differ compared to reported GBS series during the pre-COVID-19 era [78].

Our meta-analysis has some limitations that need to be taken into consideration. Firstly, the heterogeneity of the included prevalence studies was high and therefore our findings should be interpreted with caution and confirmed with a wider meta-analysis should new prevalence studies be published in the future. Excluding the prevalence finding, the remainder of our results are based on isolated published case reports or small case series. This carries a significant publication bias which may undermine the validity of the results. We opted to include those papers in our analyses, however, as they gave valuable information about the neurophysiological type, clinical course, and outcome of the cases. Moreover, although the literature was rich regarding GBS cases after the vector and mRNA vaccines, only a single case of GBS following a protein vaccine was reported. Therefore, our review could not provide a high level of evidential information about protein-vaccine-related GBS.

## Conclusions

In conclusion, vaccination against COVID-19 seems to increase slightly the risk of GBS. GBS following vaccination did not differ in characteristics from GBS during the pre-COVID-19 era. The finding that the interval between the vaccination and GBS was shorter in patients who were vaccinated with the mRNA vaccine merits further investigation in future research projects. However, it should be underscored that a possible explanation is that in the published cases of having received an mRNA vaccine, GBS was an incidental diagnosis following and not a complication related to the vaccination.
